# Processing–Microstructure–Performance Relations in Thermoformed Auxetic Hyperelastic Foams with Enhanced Energy Absorption Capacity

**DOI:** 10.1021/acsapm.5c02409

**Published:** 2025-10-11

**Authors:** Bably Das, Brett Boyle, Matthew Leoncini, George Youssef, Behrad Koohbor

**Affiliations:** † Department of Mechanical Engineering, 3536 Rowan University, 201 Mullica Hill Rd., Glassboro, New Jersey 08028, United States; ‡ Department of Physics, Rowan University, 201 Mullica Hill Rd., Glassboro, New Jersey 08028, United States; § Experimental Mechanics Laboratory, Mechanical Engineering Department, 7117 San Diego State University, 5500 Campanile Drive, San Diego, California 92182, United States; ∥ Advanced Materials & Manufacturing Institute, Rowan University, 201 Mullica Hill Rd., Glassboro, New Jersey 08028, United States

**Keywords:** Auxetic, Hyperelastic foam, Energy absorption, Thermoforming, Poisson’s ratio

## Abstract

Auxetic (negative Poisson’s ratio) foams with reentrant cell structures exhibit enhanced mechanical properties such as superior strength, energy absorption, and fracture resistance, compared to their nonauxetic counterparts. A well-established method for inducing auxeticity in cellular solids involves permanently changing the cell ribs that are buckled under compressive loads. This permanent change can be achieved by heating a deformed foam for a specific duration. In this study, a thermoforming process is developed to convert closed-cell hyperelastic polyurea foams into auxetic structures. The approach relies on rationally identifying critical compression ratios by assessing key mechanical performance attributes of the pristine foam. Auxetic transformation is achieved by applying compressive strains exceeding a defined threshold, with lateral confinement provided by a custom-designed thermoforming die. Microstructural observations and mechanical testing, including stress–strain and Poisson’s ratio measurements, confirm the successful auxetic transformation in the foam. Notably, the transition occurs at compression ratios near the nominal densification strain of the original foam. The resulting auxetic foams demonstrate negative Poisson’s ratios approaching −0.6 and exhibit energy absorption capacities several times greater than those of the pristine foam. The simplicity and scalability of the proposed thermoforming method underscore its potential for broader application in the development of the next-generation energy-absorbing structures.

## Graphical abstract

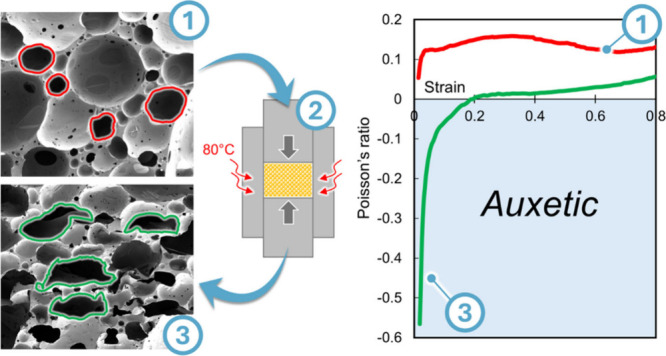

## 1 Introduction

Architected auxetic structures have garnered considerable attention in various engineering applications due to their unique combination of desirable mechanical properties, including enhanced impact energy absorption and resistance to indentation and fracture.^1–3^ This combination of desired performance attributes originates from the unique microarchitecture of auxetics, which enables the structure to contract laterally when subjected to compressive loads and vice versa, i.e., expand laterally when elongated.^4^ Such unconventional mechanical behavior is manifested at the macroscale by a negative Poisson’s ratio.^5,6^

Auxetic behavior can be implemented in architected lattice structures through the rational design of internal structure. For instance, the conversion of regular hexagons to re-entrant hexagons has been shown to activate auxeticity in honeycombs fabricated by additive manufacturing.^7,8^ In these structures, the concave topology of the re-entrant hexagons enables the cells to deform by collapsing inward when subjected to compressive loads.^8,9^ The inward collapse causes the structure to densify rapidly, thereby resisting further deformation while showing negative Poisson’s ratios.^10,11^ Such strategic manipulation of the unit cell structure has led to decades of research aimed at designing various architectures, developing new manufacturing techniques, and optimizing the performance for specific applications, including biomedical,^12^ sports,^13,14^ automotive,^15,16^ and aerospace industries.^17,18^ In recent years, in line with advancements in the design and manufacturing of flexible, multifunctional structures with tailorable mechanical and shape-morphing properties, the applications of auxetics have expanded to include soft robotics, flexible electronics, and shape-reprogrammable structures.^19–23^

An overwhelming majority of the work conducted on auxetics in recent years has been devoted to the development of ordered architected lattices. This is indeed expected as these classes of materials can be precisely tailored and custom-fabricated for a sought-after application via novel additive manufacturing techniques. However, the ability to transform an existing nonauxetic material into an auxetic one is challenging due to the limitations associated with processing methods, as well as the limited flexibility in achieving the desired properties. Nevertheless, one of the earliest studies to evidence the existence of such transformations is the work of Lakes in 1987,^24^ wherein an auxetic foam was produced from a conventional nonauxetic low-density open-cell polyester foam by causing the cell walls and ribs to protrude inward, thereby generating a re-entrant structure permanently. The reported auxetic foams were produced by subjecting the starting polyester foam to a state of triaxial compressive stress, followed by a thermal treatment. This auxetic transformation process led to a decrease in Poisson’s ratio from 0.4 to −0.7, and no significant change in the material’s Young’s modulus (71 to 72 kPa pre- to post-transformation, respectively). The decrease in Poisson’s ratio leads to a significant reduction in the material’s apparent bulk modulus, causing it to become highly compressible. A highly compressible material can be particularly useful in applications that require the absorption of large amounts of input energy, such as in impact-mitigating protective structures.^25–27^

Since the pioneering work of Lakes in 1987,^24^ numerous studies have employed similar ‘thermoforming’ processes to convert commercially available nonauxetic foams into auxetic counterparts.^28–33^ For example, Zhang et al.^28,29^ conducted comprehensive research to transform open-cell polyurethane foams into auxetic structures by subjecting them to various combinations of compression ratios and processing temperature–time conditions. Their developed uniaxial thermoforming process was shown to enable the auxetic transformation in thick foam blocks, concurrently generating negative Poisson’s ratios and increased stiffnesses. Similar results have been obtained through the thermoforming of low-density open-cell foams, as reported in Zhang et al.,^30^ showing the development of Poisson’s ratios as low as −1, as well as the anisotropy of the mechanical properties after thermoforming. In a recent study conducted by Athanasiadis et al.,^31^ the thermoforming of open-cell polyurethane foams was also investigated, resulting in a significant increase in fracture toughness of the material.

Unlike the numerous reports on the processing and characterization of auxetic open-cell foams, generating auxeticity in closed-cell foams is less established. As such, the studies on this topic are both rare and often inconclusive. One of the earliest reports on auxetic transformation in closed-cell foams is by Martz et al.,^32^ who demonstrated that a re-entrant cell topology could be induced in low-density polyethylene (LDPE) foams by applying triaxial compressive loads near the softening temperature of the material. The required hydrostatic pressures were estimated as the sum of the internal gas pressure within the closed cells and the stiffness of the solid cell walls. In their study, LDPE foams were processed for 10 h at 75 °C under hydrostatic pressures of 360 kPa. Following the heating stage, pressurization was maintained at room temperature for an additional 6 h, resulting in a total processing time of 16 h. An alternative vacuum-based process was also developed in this work. When combined with appropriate heating, the use of both hydrostatic and vacuum pressures successfully transformed LDPE into an auxetic foam. However, the strongest auxetic effect was observed when the foam was subjected to high hydrostatic pressure at 110 °C for 10 h. Similar processing attempts on closed-cell polymethacrylimide (PMI) foams, however, did not result in an auxetic transformation. In a later study, Brandel and Lakes^33^ processed various very low-density polyethylene foams using thermo-mechanical methods aimed at generating negative Poisson’s ratios and re-entrant cell topologies. Unlike Martz et al.,^32^ they employed higher processing temperatures (160 °C) but significantly shorter treatment times. The resulting foams exhibited negative Poisson’s ratios under both tensile and compressive loading, with minimum values of approximately – 0.6 recorded at very low compressive strains. Overall, these early investigations demonstrated that auxetic transformation of closed-cell foams was possible. However, the studies were primarily limited to LDPE foams, and some of the reported processing conditions involved long treatment times and elevated temperatures.

Steaming (steam penetration and condensation) processes, as reported in Duncan et al.^34^ and Fan et al.,^35^ were proposed and successfully applied to convert closed-cell polyethylene foams into auxetic foams. Subjecting a closed-cell foam to pressurized steam allows the steam to penetrate inside the cells, causing them to shrink inward as it condenses. The negative pressure caused by the condensation led to the generation of re-entrant cell shapes. Although successful in transforming conventional foams into auxetic ones, steaming processes are not scalable and are heavily limited to foams whose base material is resistant to moisture and temperatures above 100 °C, such as polyurethane and ethylene-vinyl-acetate (EVA). An alternative method to the steaming process was proposed by Duncan et al.,^36^ which involved subjecting a closed-cell low-density polyethylene foam to 100 °C at a pressure of 400–700 kPa for 6 h. The produced auxetic foam shrank by a factor of 2 to five and showed Poisson’s ratios as low as −0.2. Despite its advantages over the steaming processes in terms of the absence of humidity, the latter method is laborious, requires relatively high temperatures, and the resultant Poisson’s ratios are still far from the thermodynamic limits of −1. Considering the processing methods developed thus far for auxetic transformation in closed-cell foams, the established approaches appear to be limited to specific materials, demand specialized equipment, and often involve lengthy processing times. While these challenges likely arise from the complex gas–solid interactions inherent to closed-cell foams, the development of newer and simpler methods will be essential to further advance research in this area.

In this study, we present a simple and effective method for transforming hyperelastic polyurea foams into auxetic structures. The polyurea foams used here are inherently tough and highly energy-absorbing in their natural, nonauxetic state.^37–39^ We show that the auxetic transformation enables precise tuning of the mechanical properties and energy absorption capacity of these foams. The core idea of this work is to establish a methodology that can be extended to other foam systems, allowing similar property tailoring without the need for lengthy processing steps or specialized equipment. Our results indicate that the confined compression applied to semiclosed-cell foams to overcome the energy barrier of auxetic transformation must be selected with consideration of the foam’s native mechanical behavior. We also demonstrate that this approach enables achieving Poisson’s ratios that approach thermodynamic limits, while simultaneously enhancing the foam’s energy absorption capacity.

## 2 Modeling-Informed Design of Thermoforming Dies

As mentioned earlier, most recent research on the processing of auxetic foams via thermoforming has been conducted on open-cell foams by using uniaxial compression. While the outcomes of these studies are promising and confirm the successful generation of auxeticity, the open-die concept used may not be ideal for closed-cell foams. Closed-cell foams (and their idealized honeycomb replicas^39–41^ ) are known to develop an early stage of strain localization, which appears at the macroscale in the form of narrow shear bands with a bandwidth of approximately 1.5 times the cell diameter.^42,43^ The occurrence of such strain localizations results in a significantly heterogeneous distribution of strain in the solid portions of the foam, while also causing a limited number of cells to undergo enormous shear deformations. In contrast, the rest of the cells remain nearly strain-free. These two conditions are detrimental to a successful thermoforming practice, as the process requires as homogeneously distributed local strains as possible and the formation of re-entrant cells instead of sheared ones.

The most viable approach to prevent shear banding during the compression of closed-cell foams is to use triaxial compression, requiring complex equipment and instrumentation.^2^ A practical alternative to triaxial compression is the application of lateral confinements to an axially compressed foam piece. The advantages of confined compression on closed-cell foams were studied in this here through finite element simulations conducted on idealized cellular solids. Information regarding the finite element model and the simulation conditions are provided as Supporting Information.

As shown in Figure S1 in Supporting Information document, two loading scenarios were considered. First, the cellular solid model was subjected to uniaxial compression with no lateral confinement. In the second scenario, the same model and loading conditions were supplemented by boundary conditions that restricted the free lateral motion of the model at its side vertical edges. Comparing the simulation results for the two loading scenarios, it is clearly shown that the unconstrained loading condition leads to significant shear banding and strong localization of shear deformation in cells located within the shear band. The cells outside of the sheared area remain virtually strain-free. In contrast, applying lateral constraints to the deforming body suppresses macroscopic shear band formation, causing the strain distribution to be more homogeneous throughout the solid, and aids in the convex-to-concave transformation of cell geometries, noting again that such geometric variations are a prerequisite for auxeticity. Therefore, considering the favorable aspects of laterally constrained uniaxial compression, we designed a thermoforming die in which the foam compression was conducted in a fully constrained way. The auxetic transformation fixture design, i.e., thermoforming die, is elaborated in Sec. [Sec sec3.2].

## 3 Experimental Procedure

### 3.1 Material and Sample Preparation

Polyurea foam sheets with a nominal density of 138 kg/m^3^ (determined using ASTM D792-20) and a thickness of 18 mm were slab-molded with in-plane dimensions of 300 mm × 300 mm. The foam was produced according to the process outlined in several previous publications,^44–47^ without the use of foaming agents or a thermal curing process, which is summarized here for completeness. A frothed polyurea foam slurry was prepared by vigorously and thoroughly mixing Versalink P1000 (oligomeric diamine, Evonik) and ISONATE 143L (polycarbodiimide-modified diphenylmethane diisocyanate, DOW) with deionized water using a high-speed mixer at >10,000 rpm in laboratory ambient conditions.^44^ The foam slurry was then quickly transferred to a Teflon-coated mold with a mold cavity (30 cm × 30 cm × 1.9 cm) specifically sized to achieve the desired nominal density. The foam was left to cure and set for 24 h in ambient laboratory conditions before being demolded and dehydrated for an additional 24 h under the same conditions. The final foam sheet has a thickness of ∼18 mm.

Figure 1 depicts a micrograph of the as-prepared polyurea foam, showing spherical cells with an average cell diameter of 568.7 ± 92.3 μm and an aspect ratio of ∼1, as determined by image analysis of the SEM micrograph using ImageJ software (NIH, Bethesda, MD, USA).^45–47^ Compression cubes with dimensions of 20 × 20 × 18 mm were extracted from the foam slabs using razor blades and tested to benchmark their mechanical properties before thermoforming. Larger samples with dimensions of 40 mm × 40 mm × 18 mm were also cut from the same slab for the thermoforming process.

**Figure 1:**
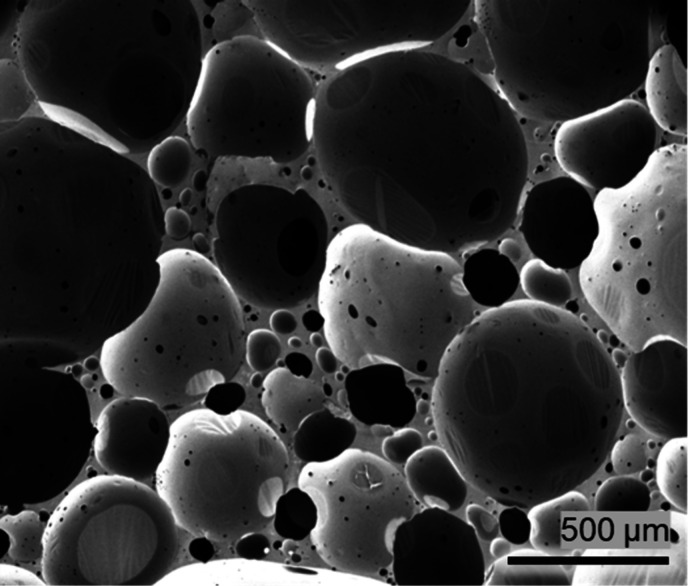
SEM micrograph of the as-received (pristine) polyurea foams.

### 3.2 Thermoforming Die Design

A fully constrained thermoforming die was designed to enable the application of the desired compression ratios on flat foam plugs. The die interior was designed to accommodate samples with in-plane dimensions of 40 mm × 40 mm. Considering the in-plane dimensions of our mechanical testing samples (20 mm × 20 mm), the die was large enough to process four samples simultaneously in each run.

Figure 2a shows the die assembly. The caps, spacers, and sleeves were made from aluminum alloy 6061. The threaded rods were stainless steel. A pristine 40 mm × 40 mm × 18 mm polyurea foam piece was first extracted from a larger foam slab and sandwiched between the two spacers (Figure 2b). The three pieces were then gently inserted into the sleeve. The spacers protruding from the sleeve allowed the foam to be positioned symmetrically inside the sleeve. The entire assembly was then sandwiched between the two cap plates. The two hex nuts on the top cap allowed controlling the desired compression ratios, while the other two hex nuts, located between the two cap plates, were used to adjust the extent of compression and ensure repeatability between subsequent thermal treatments. Two 2 mm diameter holes were drilled at the center of the front and rear walls of the sleeve for thermocouple access to monitor the temperature during thermal treatments continuously. All internal die surfaces were ground and polished to minimize friction.

**Figure 2:**
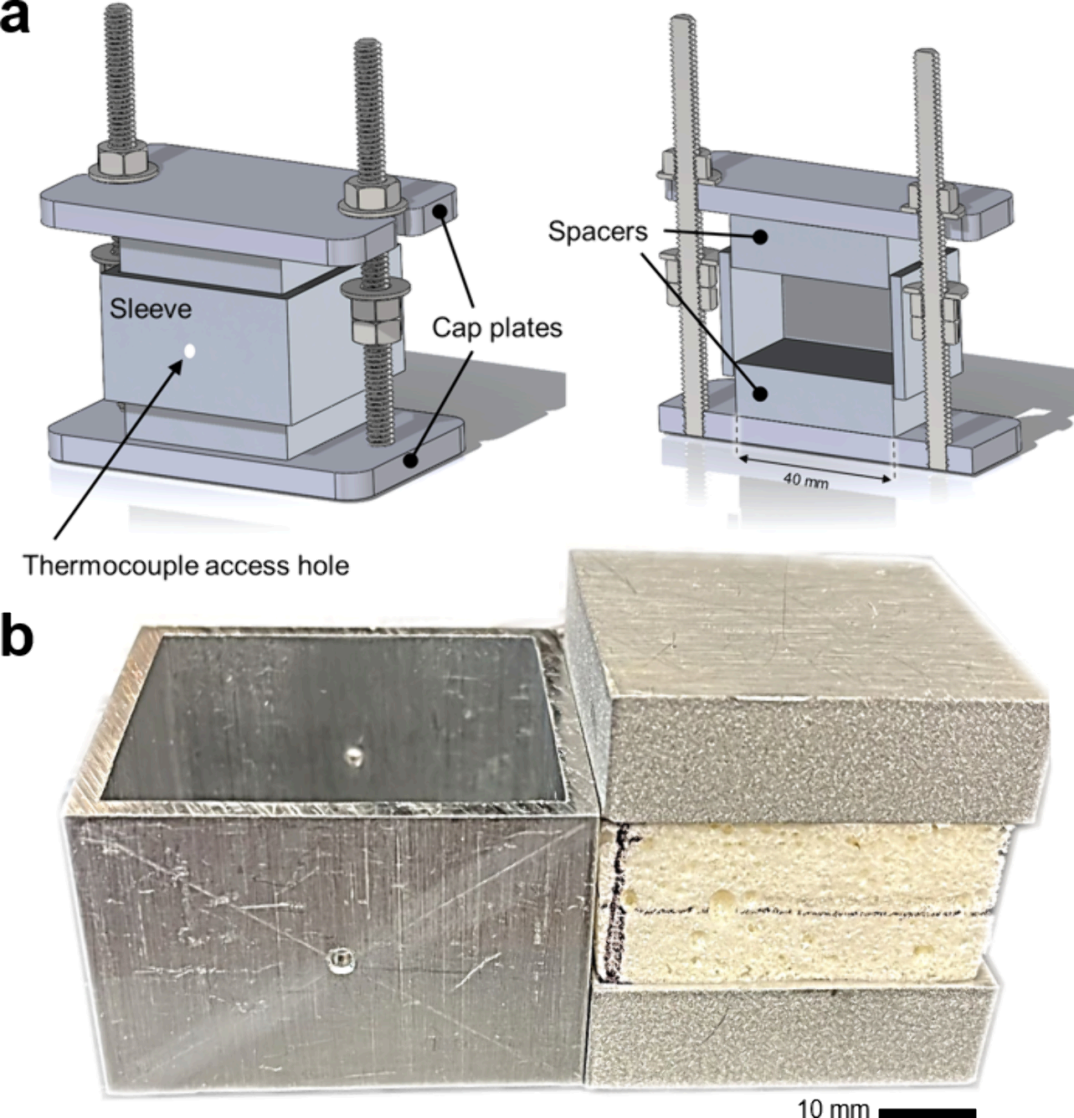
(a) The fully assembled thermoforming die showing the sleeve, cap plates, and thermocouple access hole. A sectioned view of the die showing its interior and spacers is depicted on the right. (b) Photographs of the sleeve (left) and a foam piece sandwiched between the two spacers (right).

### 3.3 Thermoforming Process

The assembled die, encompassing the compressed pristine foam, was inserted into a temperature-controlled oven (Isotemp, Fisher Scientific, MA, USA). The oven was then set to a temperature of 80 °C and turned on. As shown in Figure 3, the oven temperature ramped up rapidly to reach the set temperature within 10 min. The temperature measurements shown in Figure 3 indicate that the foam temperature stabilized at 80 °C in approximately 55 min. The assembly was kept inside the oven for 3 h, followed by removal and disassembly. Note that the total processing time of 3 h includes approximately 1 h for temperature ramp-up, followed by 2 h of soaking during which the temperature remains stable and nominally constant throughout the compressed foam sample. The processed foam piece was allowed to cool to room temperature in ambient conditions. The thermoformed foam piece was then sliced into four smaller samples. One of the four samples was used for SEM microscopy purposes. The other three were mechanically tested after 48 h of resting at room temperature. This process was repeated for several different compression ratios, as discussed next.

**Figure 3:**
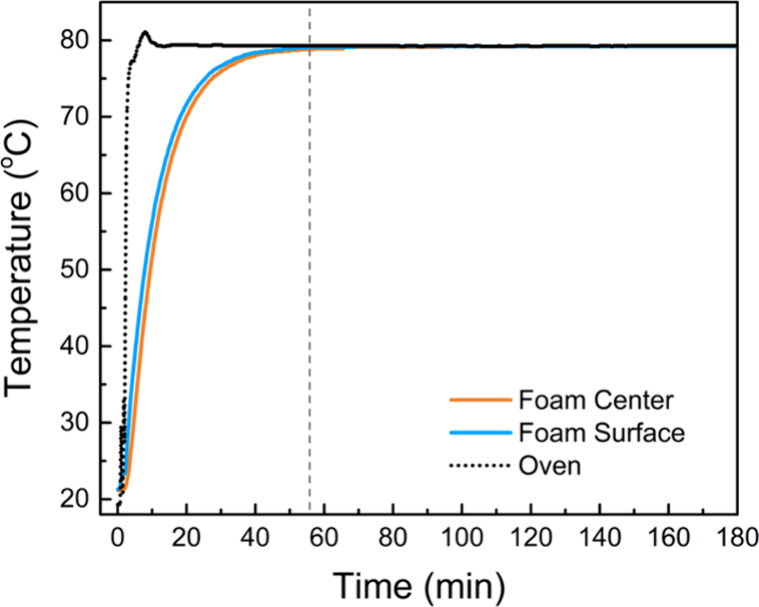
Variations of oven interior, foam center, and foam surface temperatures with time during the thermoforming process. Foam core and surface temperatures were simultaneously measured with thermocouples fed through the die via access holes on the sleeve. The dashed vertical line indicates the time when the temperature stabilizes in the foam at 80 ± 1 °C.

The processing temperature and time (80 °C, ∼2 h) were selected based on the results obtained and discussed by Uddin.^48^ It has been previously shown that the examined polyurea foams reset their prior deformation history when subjected to thermal treatments under the aforementioned processing conditions. It is also imperative to note that the glass transition temperature of the polyurea foam examined here is −50 °C,^38^ ascertaining that all thermal treatments discussed were performed above *T*_*g*_.

### 3.4 Selection of Compression Ratios Informed by Mechanical Tests

The critical requirement for a successful thermoforming process is the applied prestrain before thermal treatment. In the available literature, the magnitude of this prestrain, herein referred to as the compression ratio, has been predominantly chosen iteratively. Here, we formalize a procedure that can help identify the critical compression ratios for the polyurea foam.

The first two critical compression ratios selected in this work are associated with two metrics used to evaluate the energy absorption capacity in cellular solids.^49^ The first is the strain (compression ratio) at which the energy absorption efficiency is maximized. Energy absorption efficiency, *η*, is defined as the ratio between the specific absorbed energy (the area below the stress–strain curve) and the absorbed energy of a fully compressed ideal absorber,^45,50,51^ expressed in eq 1.

1 $$\eta =\frac{\int \sigma d\varepsilon }{\sigma }$$

where, σ and *ε* denote compressive stress and strain, respectively. The compressive strain that maximizes the above metric is also regarded as the densification onset strain, *ε*_*d*_, in a cellular solid.

The second critical compression ratio is associated with the point at which the energy absorption ideality metric is maximized. The ideality, *I*, is defined as the ratio between the specific absorbed energy of real and ideal foams loaded to the same strains, as expressed in eq 2.^49–51^

2 $$I=\frac{\int \sigma d\varepsilon }{\sigma \varepsilon }$$

The engineering stress–strain curves for the pristine (before thermoforming) foams were measured by subjecting 20 mm × 20 mm × 18 mm samples to uniaxial compression at a constant crosshead speed of 5 mm/min, equivalent to a nominal strain rate of 0.0046 s^–1^. All mechanical tests were performed using a Shimadzu AGS-X test frame equipped with a 10 kN load cell and operated in displacement-control mode.

To measure the apparent Poisson’s ratio of the foams in pristine and thermoformed conditions, all mechanical tests performed herein were accompanied by 2D digital image correlation (DIC) analyses. A single 5-megapixel camera fitted with a 100 mm macro lens was set up in front of the deforming foam. The camera-facing surface of the foam sample was sparsely sprayed with a matt black paint. The image contrast, amplified by the natural pore structure of the white foam and the applied black paint, provided sufficient contrast for successful image correlation analyses. All image correlation analyses were performed using subset and step sizes of 101 and 20 pixels, corresponding to 1.28 mm and 250 μm, respectively. The subset sizes were intentionally selected to be large enough to encompass at least two cells. The associated strain noise floor was estimated to be 550 × 10^–6^, determined using the procedures elaborated in Koohbor et al.^52^ Due to the large deformation nature of the experiments performed here, an ‘incremental’ correlation method was used.^53^ Longitudinal, *ε*_*yy*_, and transverse, *ε*_*xx*_, strain fields extracted from DIC analyses were spatially averaged. The ratio between the two strain values at each image was determined as the nominal Poisson’s ratio of the foam. The secant (simple ratio) definition of Poisson’s ratio was preferred over its tangential (ratio of strain increments) definition in this work. As discussed in detail by Koumlis and Lamberson,^54^ the use of the secant definition underestimates the negative Poisson’s ratios in auxetic foams. Therefore, if Poisson’s ratio is found to be negative based on the secant formulation, it ensures that the foam has indeed transformed into an auxetic one.

While Poisson’s ratio measurement was necessary to evaluate the degree of auxeticity in pre- and post-thermoformed foams, its variation with strain in pristine foams was also used to identify the third critical compression ratio in this work.

## 4 Results and Discussion

### 4.1 Mechanical Behavior of Pristine Foam and Critical Strains

As the first step to show the thermal stability of the pristine foams and to confirm that the thermal treatment in the absence of mechanical compression does not lead to any significant change in the utilized foam. The load-free polyurea foams were subjected to the same thermal treatment used later during the thermoforming process, i.e., 3 h at 80 °C in the same thermoforming die but without any compression ratio. Figure 4 shows the engineering stress–strain and the corresponding Poisson’s ratio-strain curves for polyurea foams before and after load-free thermal treatment, indicating no discernible variations between the two conditions. The slight difference between the pristine and load-free annealed samples indicates (1) the removal of mold compression locking and (2) the thermal stability of polyurea foams up to 80 °C. The variation of Poisson’s ratio with compressive global strain is shown in Figure 4b, illustrating a persistent local minimum at a strain of 0.7 due to the collapse of the unit cells.

**Figure 4:**
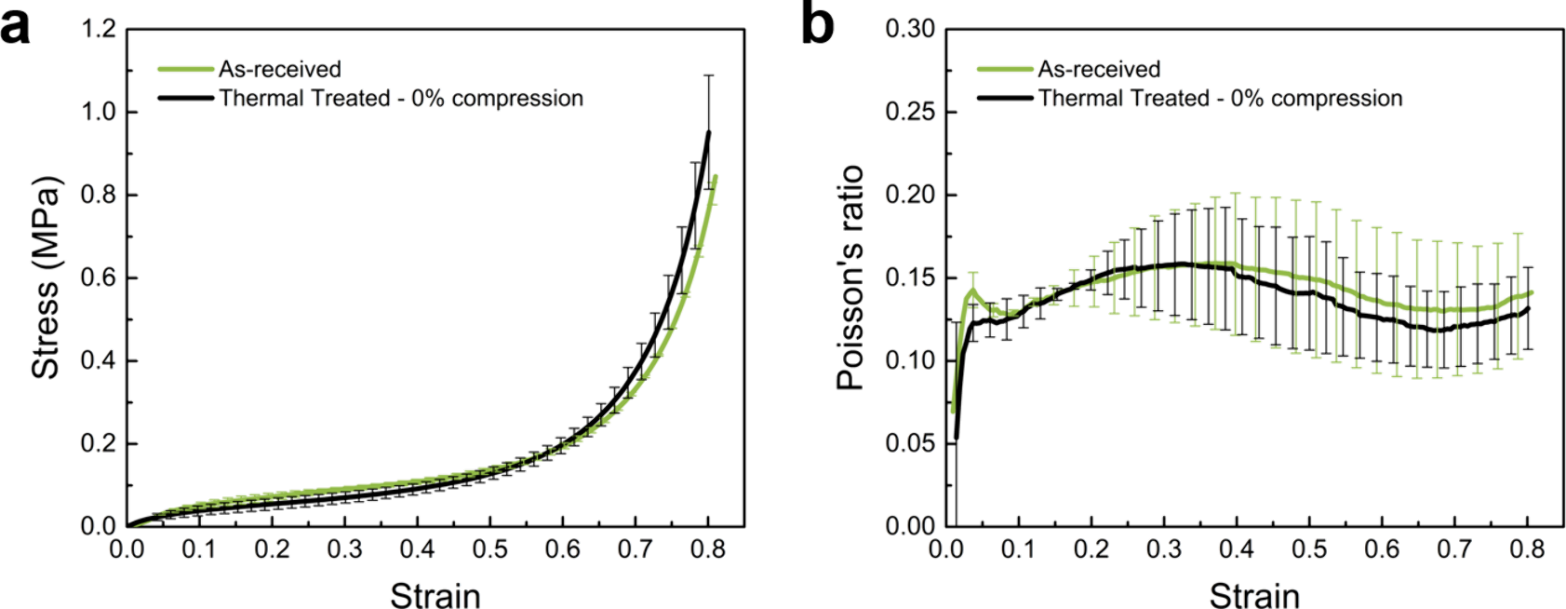
(a) Stress–strain and (b) Poisson’s ratio–strain curves for polyurea foams in pristine and load-free thermally treated conditions. Scatter bars represent variations across three separate measurements.

Because it was shown that the thermal treatment at 0% compression did not result in any discernible variations in the mechanical properties of polyurea foams here, henceforth we report and use all measured values for such conditions, i.e., the control conditions hereafter will be the foam thermally treated for 3 h at 80 °C and a 0% compression ratio. Figure 5a isolated the average engineering stress–strain curves (*n* = 3) of the load-free thermally treated polyurea foams. The stress–strain response of the foam exhibits a behavior typical of hyperelastic foams, characterized by a linear region, followed by an extended stress plateau and a steep stress increase that marks the onset of densification. Figure 5b and Figure 5c show the corresponding energy absorption efficiency and ideality metrics as a function of axial compressive strain. The efficiency plot (Figure 5b) indicates a distinct peak at a compressive strain of ∼0.5, marking the onset of macroscopic densification. The ideality (Figure 5c) peaks at compressive strains of ∼0.25. Hence, the respective strains to maximum efficiency and ideality were considered as two critical compression ratios to use in the thermoforming processes. Interestingly, the manifestation of cell collapse at 70% compressive strain (based on the Poisson’s ratio in Figure 4b) points to another critical compression ratio, which was also employed in the auxetic transformation process.

**Figure 5:**
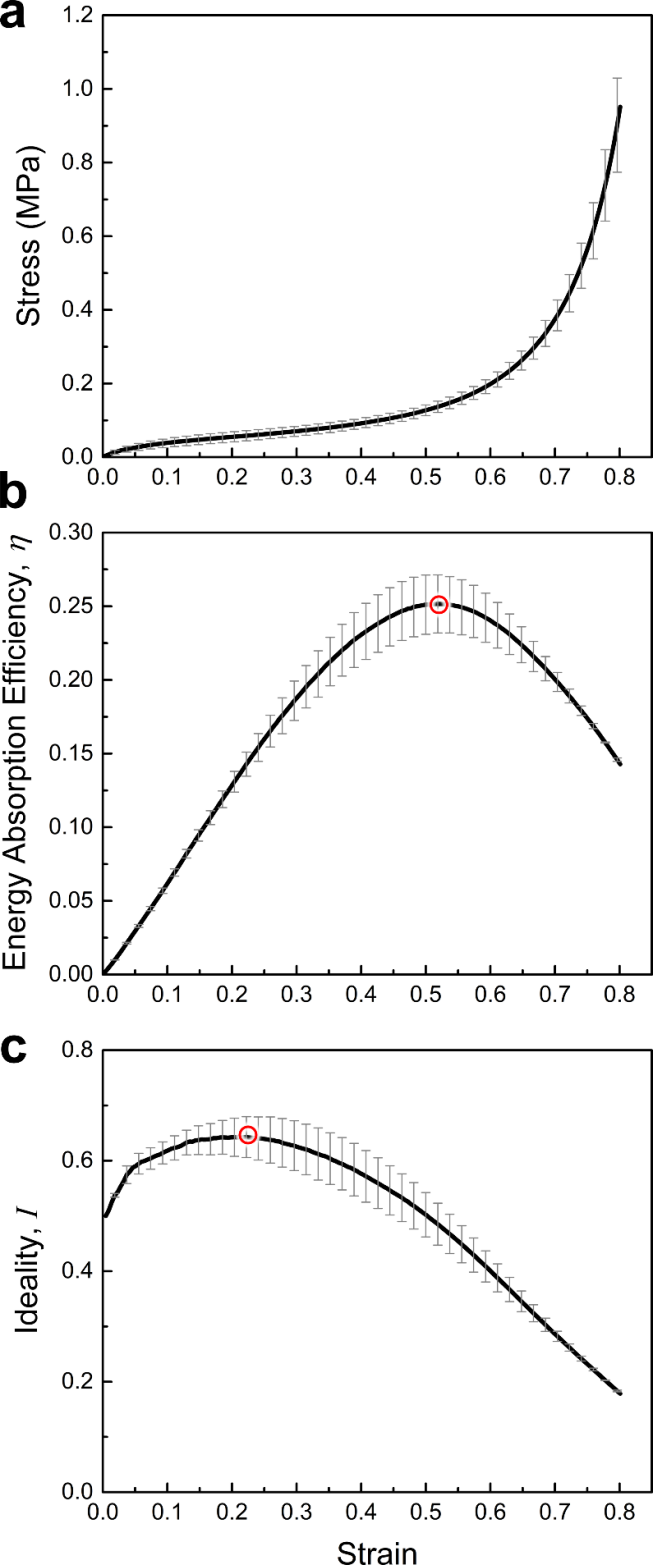
Variation of (a) stress, (b) energy absorption efficiency, and (c) ideality with strain for the control foam. Hollowed red circles in (b) and (c) mark the maxima on each curve.

The apparent Poisson’s ratio of the control foam was also measured using digital image correlation. Figure 6a shows the distribution of longitudinal and transverse strain fields across the front surface of the foam at different compressive strains. Both strain fields show a nonuniform distribution across the field of view with no particular spatial trends, except that the *ε*_*xx*_ maps appear to have some large positive strain areas that are preserved over the entire range of compressive strains. In contrast, *ε*_*yy*_ fields show nonuniform distribution at the beginning of the compression; however, the degree of nonuniformity appears to mitigate at larger global strains. The initially higher levels of strain nonuniformity are consistent with previous observations^55^ and are likely caused by the material and cell-scale heterogeneities. Applying larger global strains causes the compression and complete collapse of cells everywhere, thereby showing a progressive increase in uniformity of the strain fields. The associated numerical values for the foam’s Poisson’s ratio were extracted as the ratio of the in-plane strain components, averaged over the entire area of interest. Variation of Poisson’s ratio with compressive global strain is shown in Figure 6b. As elucidated in this figure, the pristine polyurea foams tested here consistently showed a slight drop at 0.7 strain. The decrease in Poisson’s ratio observed in the 70% strain range is consistent with previous reports on other closed-cell polyurea^56^ and polyethylene^33^ foams. This trend is attributed to the progressive flattening of the cells under axial compression, which reduces the rate of outward expansion as compressive axial strain increases. Once global densification strain is reached, cell flattening becomes saturated, and the compressed foam begins to resemble the behavior of its nonporous solid constituent (i.e., polyurea), exhibiting a larger Poisson’s ratio. Therefore, the presence of a local drop in the value of Poisson’s ratio is linked to the completion of the densification process, wherein the majority of spherical cells in the foam collapse into flattened ellipsoidal shapes.

**Figure 6:**
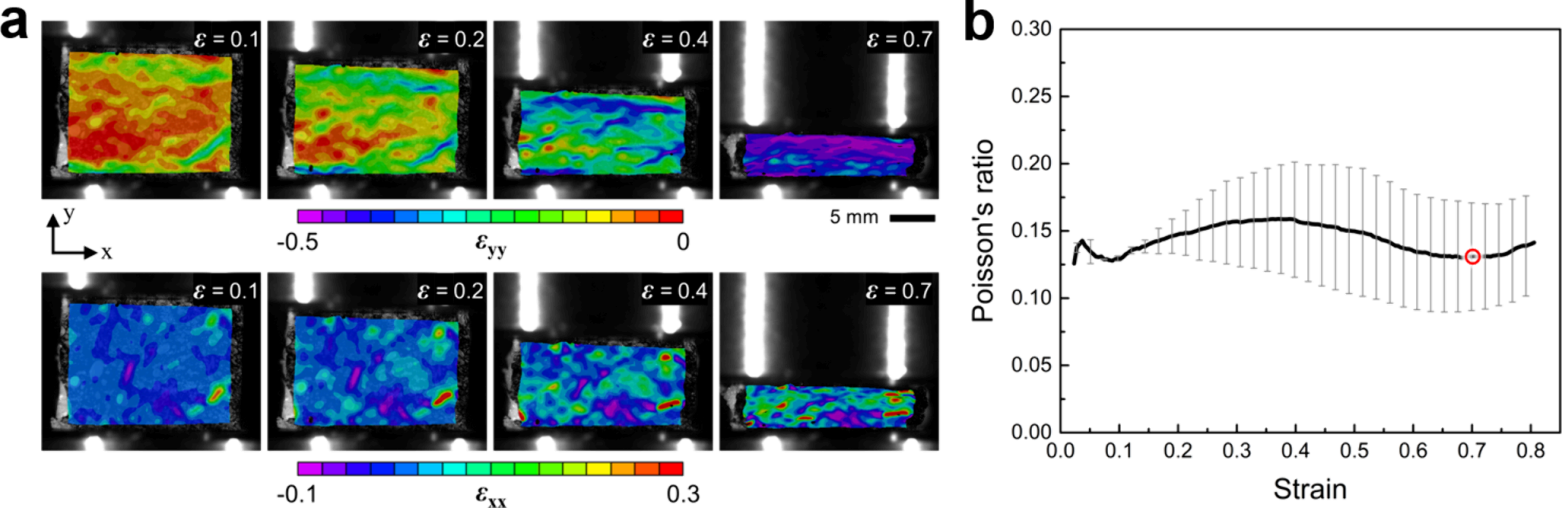
(a) Contour maps showing the evolution of longitudinal, *ε*_*yy*_, (top row) and transverse, *ε*_*xx*_, (bottom row) strain fields at different global strains, *ε*. (b) Variation of Poisson’s ratio with strain for the control conditions, i.e., the thermally treated foam at 0% compression. The local Poisson’s ratio minimum is marked with a hollowed red circle.

### 4.2 Microstructural Developments after Thermoforming

SEM micrographs of the thermoformed foams are depicted in Figure 7, compared to a micrograph of the control. The compression was applied in the *y*-direction in all case studies highlighted herein. These micrographs underscore the thermal transformation process as a function of axial compression, where the nearly spherical unit cells (Figure 7a) of the control samples begin to transform into ellipsoids at the 30% compression ratio. Some cells in the 30%-compressed thermoformed cells also exhibit small wrinkles on their exterior, indicating the permanent microscale buckling that occurred at the cellular scale. Similar microcellular evolution also occurred in the case of samples with a 50% compression ratio. However, the cell structure of the 70% compression ratio samples exhibited a pronounced topological evolution into deformed re-entrant ellipsoids. Inwardly deformed cell walls characterize the re-entrant cell architecture. Collectively, the SEM micrographs in Figure 7 ascertain the successful thermal auxetic transformation of polyurea foams as a function of a broad range of compression ratios.

**Figure 7:**
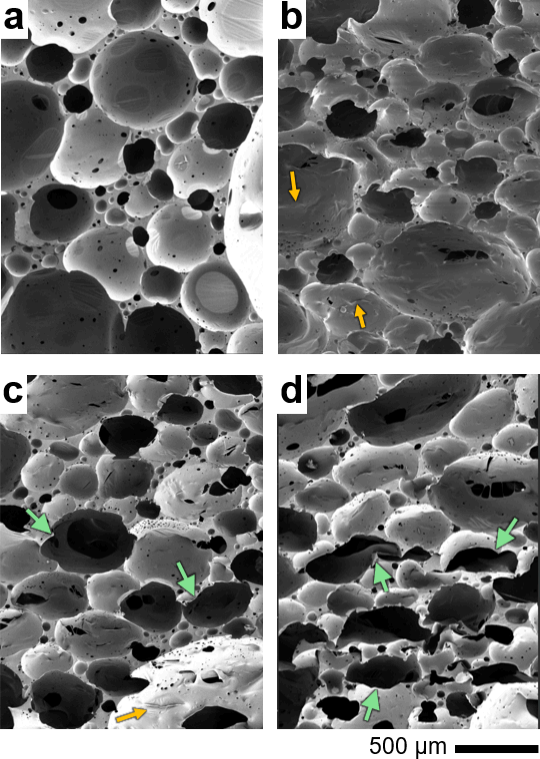
SEM micrographs showing post-thermal treatment cell structure of foam samples with (a) 0% (control), (b) 30%, (c) 50%, and (d) 70% compression ratios. Yellow and green arrows point to surface wrinkles and concave cells, respectively.

### 4.3 Mechanical Behavior of Thermoformed Foams

Uniaxial compression tests were conducted to evaluate the impact of thermal auxetic transformation on the mechanical properties of polyurea foams. Figure 8a shows the engineering stress–strain curves obtained from thermoformed samples as a function of compression ratio, compared to the behavior of the control foam. Irrespective of the compression ratio, all foams retain their general nonlinear stress–strain curves, exhibiting a positive correlation between the mechanical load-bearing capacity and the compression ratios used during the thermal transformation process. The increase in load-bearing capacity is potentially attributed to the transformations from spherical to ellipsoid and from convex to concave (reentrant). That is to say, auxetic polyurea foams leveraged the mechanical behavior of the base material more effectively than their pristine counterparts, given the predeformed cells during thermal transformation, i.e., higher stress values at higher global strains.

**Figure 8:**
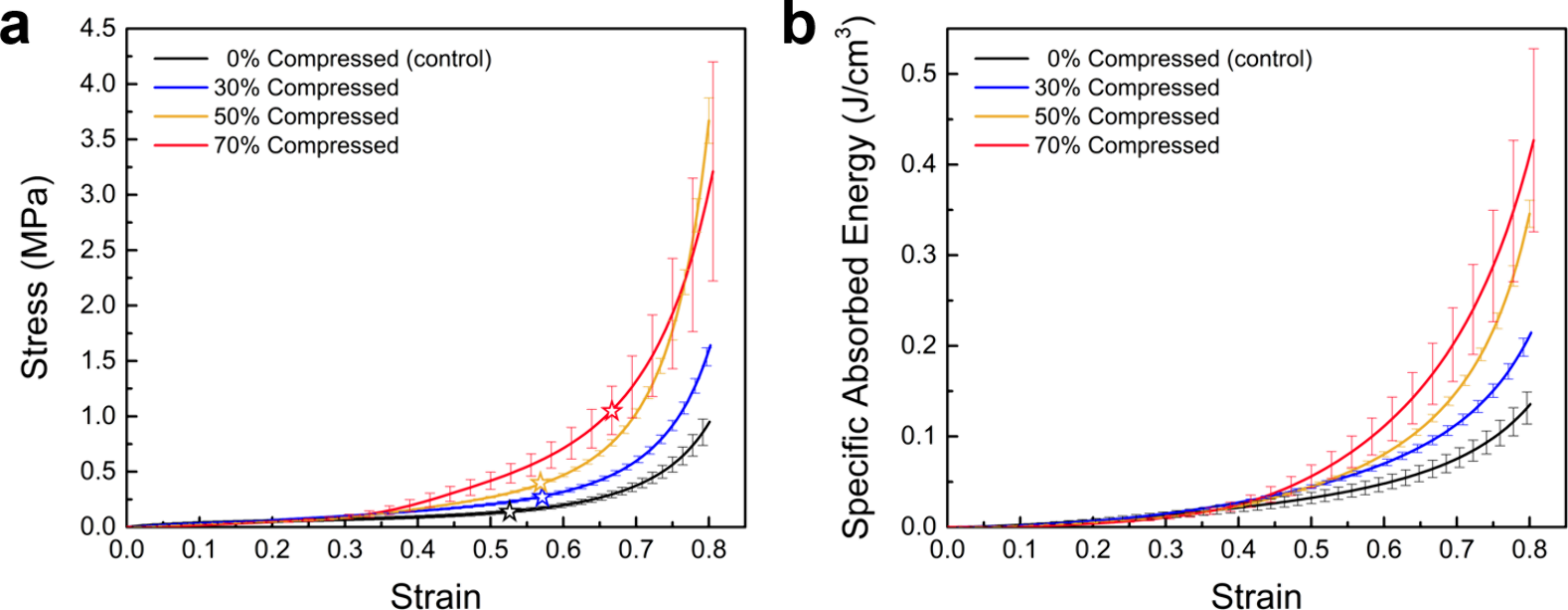
(a) Stress–strain curves and (b) variation of specific absorbed energy with strain for control and thermoformed samples at different compression ratios. Scatter bars represent experimental variations across three separate measurements. Star markers in (a) indicate the densification strain on each curve.

The apparent densification onset strain, *ε*_*d*_, for each condition was also determined using the procedure described in Sec. [Sec sec3.4] by identifying the global strain that corresponds to *η*_*max*_. The densification strains are indicated by hollowed star markers directly on the stress–strain curves in Figure 8a. The densification strains also follow a trend similar to that of strength as a function of increasing compression ratio, which is a favorable attribute for the development of next-generation impact-mitigating structures. Finally, Figure 8b shows the effect of the compression ratio on the specific absorbed energy, showing an increase in the latter at higher values than the former. For example, the results from Figure 8b indicate a 4-fold increase in the energy absorption of the 70%-compressed thermoformed sample compared with the 0%-compressed control foam.

The significant enhancement in energy absorption observed in the 70%-compressed thermoformed sample can be attributed to fundamental differences in deformation mechanisms relative to the original cell structure. In the pristine foam, macroscale deformation is primarily governed by elastic bending followed by localized buckling of cell walls. In contrast, the re-entrant topology in the thermoformed samples alters the instability mode at cell scales. Specifically, the inward-folded ribs in a re-entrant morphology undergo coordinated hinging and progressive collapse rather than isolated buckling.^41^ This mechanism delays the onset of densification and extends the plateau stress region, leading to higher strain energy absorption.

### 4.4 Poisson’s Ratio and Auxeticity

Poisson’s ratios were characterized by tracking the transverse and longitudinal strain fields by DIC. Figure 9a shows in-plane strain contour maps for thermoformed foam samples at different global strains. Specific differences and characteristics can be identified by comparing the DIC results of the thermoformed samples. First, the initial height of the samples appears to be shorter for higher compression ratios, since thermoforming causes permanent axial deformation. Specifically, sample thickness measurement for the thermoformed samples compressed at 30%, 50%, and 70% compression ratios indicated 13.8, 10.31, and 8.9 mm, respectively. Second, there is the presence of strain nonuniformity in all thermoformed conditions and at all levels of global strain. However, the degree of strain nonuniformity appears to decrease at larger compressive strains due to closure of the cells. Third, all *ε*_*xx*_ maps show largely negative strain regions, with the degree of negative strain increasing in thermoformed samples processed at higher compression ratios, evidencing the successful auxetic transformation.

**Figure 9:**
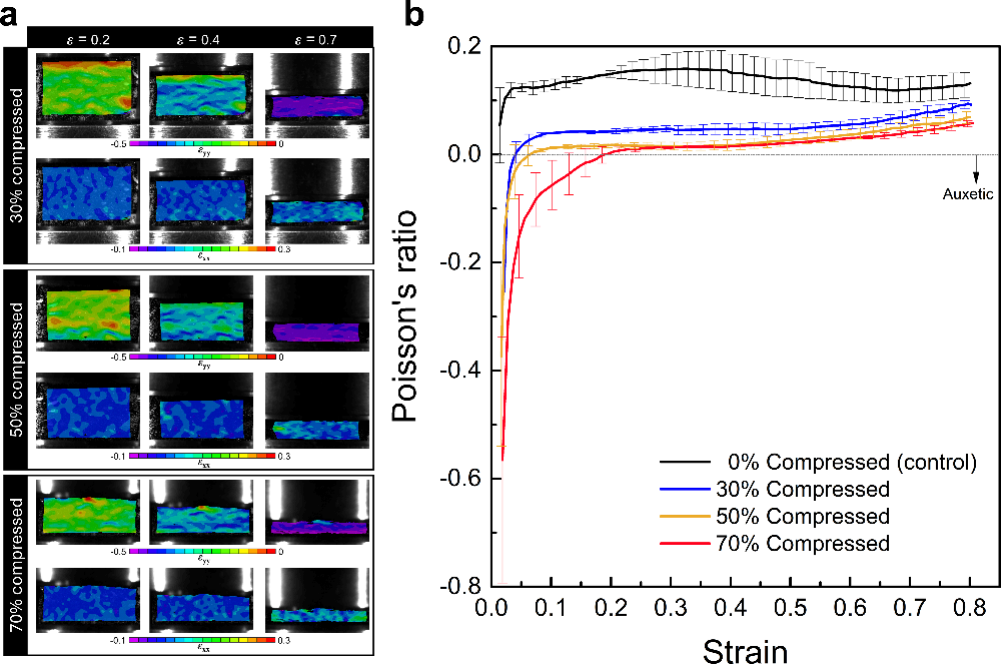
(a) Contour maps showing the evolution of longitudinal, *ε*_*yy*_, and transverse, *ε*_*xx*_, strain fields for thermoformed foams at different global strains, *ε*. (b) Variation of Poisson’s ratio with strain for the thermoformed foam samples.

Poisson’s ratios vs. the axial strains are plotted in Figure 9b for different compression ratios. The control samples exhibit a positive Poisson’s ratio over the entire strain range. Increasing the compression ratio to 30% causes a noticeable shift in Poisson’s ratio to lower values, although Poisson’s values remain broadly positive. Further increase in the compression ratio led to a slight decrease in Poisson’s values, crossing the auxeticity threshold at very small strains. This trend continues for the 70%-compressed foam, exhibiting an evident shift toward auxeticity at strains below 0.2. These observations confirm that the thermoforming process facilitates auxetic transformations in the foam at compression ratios exceeding 50%, i.e., compressions that exceed the nominal densification strain of the foam. Another noteworthy observation is the minimal variations in Poisson’s ratios for the thermoformed foams at strains above 0.25, irrespective of the compression ratio, due to higher engagement of the deformation mechanisms of the base materials and the relative sliding between the collapsed cells.

### 4.5 Process–Property Maps

Based on the preceding results and discussion, the energy absorption and Poisson’s ratios are contextualized in terms of the thermoforming parameters, i.e., the compression ratios. On the one hand, higher compression ratios result in considerable auxeticity at strains ranging from 0 to 0.25. On the other hand, the energy absorption capacity in thermoformed foams exhibits a significant increase at strain ranges greater than 0.35, noting that none of the samples examined here displayed auxeticity within these strain ranges. The process-property map shown in Figure 10a visualizes the interplay between strain-dependent energy absorption and Poisson’s ratio. While the overlap between the favorable range of the two parameters (i.e., negative Poisson’s ratio and high specific energy absorption) does not appear to be readily achievable at all strain conditions, the premise that these properties are tailorable as a function of the compression ratio is promising. Notably, the energy absorption capacity of the thermoformed foams increases significantly as the foam approaches its densification limit. This finding emphasizes that a practical approach to improving the energy absorption capacity of thermoformed closed-cell foams will require compression ratios that are, at a minimum, very close to the nominal densification strain of the foam in its strain-free, pristine condition.

**Figure 10:**
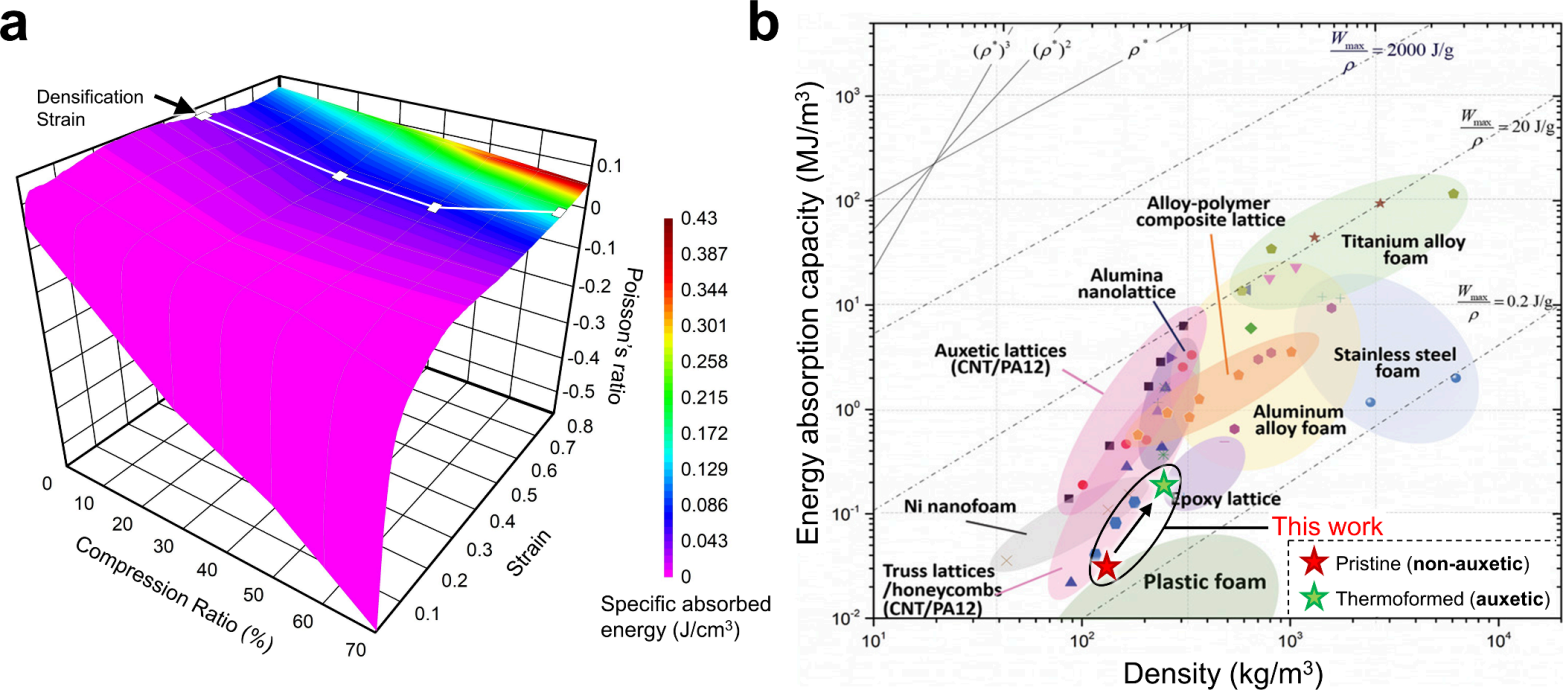
(a) Process–property map showing the interdependence of compression ratio, Poisson’s ratio, energy absorption, and strain on thermoformed polyurea foams. The densification strain locus is marked by a solid white line. (b) Ashby map of specific energy absorption (energy absorption per unit volume) vs. density for various classes of stochastic (foams) and ordered (lattices) cellular structures. Pristine and 70%-compressed thermoformed polyurea foams discussed in this work are included in the map. Energy absorption capacities for thermoformed foams are determined at their densification strain. Original map reported in Yuan et al.^57^ and reproduced here with permission. Copyright 2019 Wiley.

At the outset, Figure 10b shows an Ashby map of specific energy absorption versus density for a wide range of polymeric and metallic foams and lattice structures, including the results from this research study, i.e., pristine and thermoformed polyurea foams. The nonauxetic pristine foam falls within the category of polymeric foams. Interestingly, the higher energy absorbing thermoformed derivatives of the polyurea foam move well outside of this category and toward epoxy lattices. It is indeed true that a successful implementation of the thermoforming process increases the density of the foam, as evidenced by the permanently reduced thickness of the foams after thermoforming. However, the gain in the energy absorption capacity dominates the unfavorable density increase, leading to the development of materials that extend the envelope of polymeric foams to higher limits. Such developments must be realized not only in terms of the property improvement, but also in terms of the manufacturability and scalable production of these materials. Fabrication of auxetic structures through additive manufacturing is typically constrained by several factors, including the maximum printable dimensions, limited uniformity in larger samples, and the considerable time and cost associated with high-resolution and large-scale printing. In contrast, the processing approach introduced and demonstrated in this work offers a practical, inexpensive, and scalable alternative. Importantly, the base polyurea foam used here can be produced at industrial scales with large dimensions and consistent properties, at a fraction of the time and cost required by additive manufacturing.^44^ The subsequent conversion into auxetic structures relies on a relatively simple processing methodology that requires only straightforward instrumentation, making it inherently more suitable for upscaling. While potential challenges may arise, such as maintaining uniform re-entrant topology across thicker or larger samples, and ensuring reproducibility during high-throughput processing, these are engineering issues that can be systematically addressed. For example, process optimization through controlled thermal cycles, automated handling, and continuous-feed processing lines could facilitate large-scale production. Altogether, the approach discussed in this work can be regarded as a value-adding and attractive approach to creating new protective foams with programmable behaviors for applications in the sports, automotive, and defense industries.

## 5 Conclusions

A thermoforming process was developed to convert nonauxetic hyperelastic polyurea foams into auxetic foams. Informed by finite element simulations, the thermoforming die was designed with lateral confinement to promote homogeneous strain distribution within the solid parts of the foam and facilitate the development of reentrant cell topologies during thermoforming. Compression ratios for an effective auxetic transformation were determined based on the mechanical and energy absorption metrics of the pristine foam. It was demonstrated that thermoforming at 80 °C for 2 h is sufficient to induce auxeticity in polyurea foams when confined compression ratios of at least 50% are applied.

The resulting thermoformed foams exhibited negative Poisson’s ratios as low as −0.6 in low strains, while higher compression ratios enabled the retention of auxetic behavior up to 20% strain. Additionally, the thermoformed auxetic samples demonstrated enhanced strength (higher plateau stress) and energy absorption capacity compared to their nonauxetic counterparts. In particular, foams processed at 70% compression ratios showed up to nearly 4× increase in energy absorption relative to pristine samples. These significant improvements highlight the potential of this scalable thermoforming approach for producing protective foams with programmable mechanical behaviors for diverse industrial applications.

## Supplementary Material



## Notes

Further information about FE simulations and additional SEM images of thermoformed samples (PDF)
