# Supplementary material for: Processing–Microstructure–Performance Relations in Thermoformed Auxetic Hyperelastic Foams with Enhanced Energy Absorption Capacity
Source: ACS Appl Polym Mater. 2025 Oct 11;7(20):13586–96. doi: 10.1021/acsapm.5c02409 (PMC12560077; doi:10.1021/acsapm.5c02409)
Supplement: Supplementary file 1 [file ap5c02409_si_001.pdf]

Supporting Information

Processing-Microstructure-Performance Relations in Thermoformed Auxetic Hyperelastic Foams with Enhanced Energy Absorption Capacity

Bably Das [a], *Brett Boyle* [b], *Matthew Leoncini* [a], *George Youssef* [c], *Behrad Koohbor* [a,d,*]

a. Department of Mechanical Engineering, Rowan University, 201 Mullica Hill Rd., Glassboro, NJ, 08028, USA

b. Department of Physics, Rowan University, 201 Mullica Hill Rd., Glassboro, NJ, 08028, USA

c. Experimental Mechanics Laboratory, Mechanical Engineering Department, San Diego State University, 5500 Campanile Drive, San Diego, CA, 92182, USA

d. Advanced Materials and Manufacturing Institute, Rowan University, 201 Mullica Hill Rd., Glassboro, NJ, 08028, USA

*. Corresponding Author, koohbor@rowan.edu

S1. Finite Element (FE) Simulations

The base material used in FE simulations is polyurea with ascribed constitutive behavior of the Ogden 4th-order model. An idealized array of cells was used in these 2D simulations, as it has been previously shown to provide essential information about the deformation response of the foam, especially in comparative studies and for understanding the fundamental mechanisms that govern the multiscale deformation of closed-cell polyurea foams [S1]. FE simulations were performed in the commercial software ABAQUS. The FE model was meshed with 36,515 triangular linear plane

strain (CPE3) elements (**Figure S1a**). Hard-contact surface conditions were assigned to all cells to ensure no mutual penetration between the nodes and elements of opposing cell walls at large compressions. More details regarding the FE simulations can be found in our previous publications [S1].

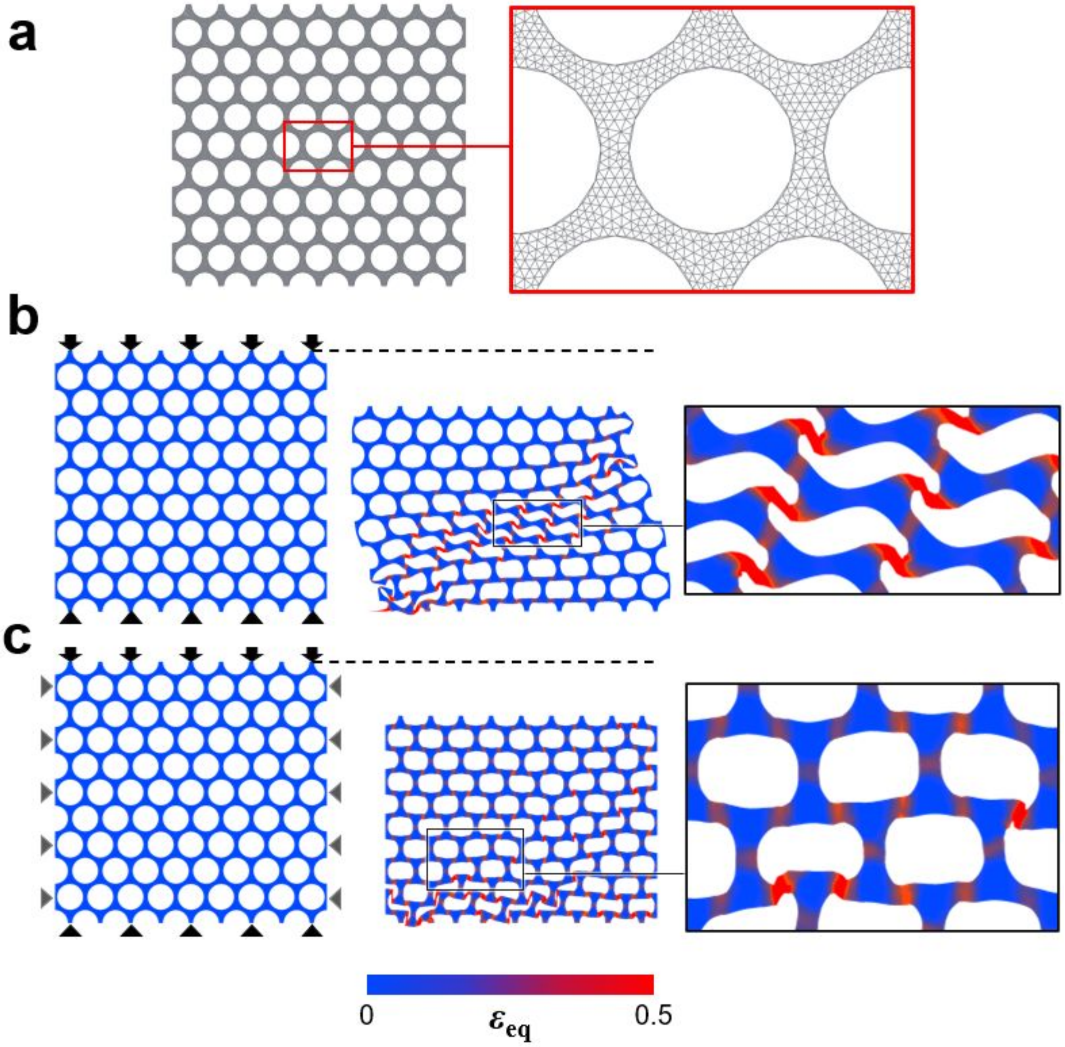

Figure S1. (a) FE model of a representative volume element of the polyurea foam with a magnified view of the mesh geometry. Initial and 20% compressed states of the foam at (b)

unconstrained and (c) laterally constrained conditions. Contour maps in (b) and (c) show the equivalent strain fields.

S2. Additional SEM Images

Additional SEM images are presented in **Figure S2** to confirm the repeatability of the results reported in the main article. These micrographs were acquired from random locations within multiple 70%-compressed thermoformed samples, each processed independently. Reentrant morphology transformation is evident in these micrographs.

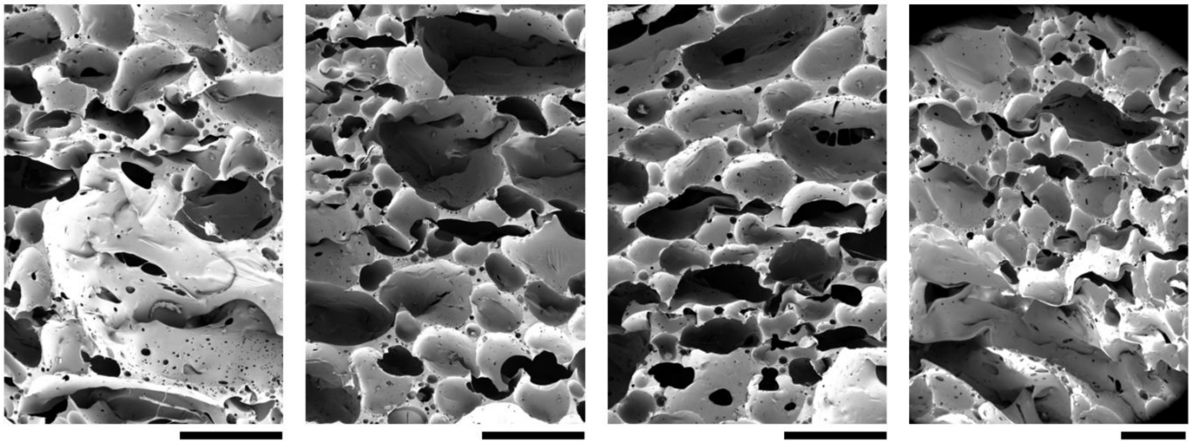

Figure S2. SEM micrographs showing thermoformed cell structure of foam samples with 70% compression ratio. These images are acquired from random locations in multiple samples processed independently. All scale bars represent 500 μm.

References

[S1] Koohbor, B.; Youssef, G.; Uddin, K.Z.; Kokash, Y. Dynamic Behavior and Impact Tolerance of Elastomeric Foams Subjected to Multiple Impact Conditions. Journal of Dynamic Behavior of Materials 2022, 8, 359–370.
